# Sleep Irregularity and Short Sleep Duration Are Associated with Less Favorable Cardiometabolic Profiles in Healthy Adults: A Cross-Sectional Study

**DOI:** 10.3390/nu17233763

**Published:** 2025-11-30

**Authors:** Sofia Lotti, Antonia Napoletano, Monica Dinu, Elisabetta Picchi, Ugo Faraguna, Ilaria Giangrandi, Francesca Cesari, Rossella Marcucci, Francesco Sofi, Barbara Colombini

**Affiliations:** 1 Department of Experimental and Clinical Medicine, University of Florence, 50134 Firenze, Italy; sofia.lotti@unifi.it (S.L.); antonia.napoletano@unifi.it (A.N.);; 2 Department of Translational Research and of New Surgical and Medical Technologies, University of Pisa, 56126 Pisa, Italy; 3 Department of Developmental Neuroscience, IRCCS Stella Maris Foundation, 56128 Pisa, Italy; 4 Unit of Nutrition, Careggi University Hospital, 50134 Florence, Italy; 5 Atherothrombotic Diseases Center, Careggi University Hospital, 50134 Florence, Italy

**Keywords:** sleep, actigraphy, cardiovascular risk, body composition, dietary habits

## Abstract

**Background/Objectives**: The aim of this study is to evaluate the relation between sleep quantity (TST), efficiency (SE) and regularity (SRI) and cardiometabolic parameters and eating habits. **Methods**: Seventy clinically healthy adults (74% females; mean age 28.3 ± 10.1 years) were recruited at the Clinical Nutrition Unit of Careggi University Hospital, Florence, between October 2023 and December 2024. Sleep was monitored for 7 days using a Fitbit Alta HR actigraphy. Cardiometabolic health was assessed via bioimpedance and blood samples. Dietary habits were evaluated through 3-day food diaries and the Medi-Lite questionnaire. **Results**: Participants had an average TST of 7.4 ± 1.1 h, SE of 84.9 ± 6.9%, and SRI of 62.2 ± 19.9. Lower SRI (≤41, 1st quintile) was associated with higher fat mass (19.9 ± 6.7 vs. 15.2 ± 6.6%), higher total cholesterol (183.9 ± 20.9 vs. 155.0 ± 26.8 mg/dL), and lower folate (3.6 ± 1.6 vs. 5.6 ± 2.5 ng/mL) compared to higher SRI (≥80, 5th quintile). Sleeping <7 h/night was linked to higher BMI (22.6 ± 2.1 vs. 21.5 ± 2.0 kg/m^2^) and homocysteine (11.4 ± 2.3 vs. 10.4 ± 3.3 μmol/L). Weak but significant inverse correlations emerged between TST and BMI (R = −0.26, *p* = 0.02) and between SRI and cholesterol (R = −0.28, *p* = 0.01), but these associations disappeared in the multivariable linear regression adjusted model. **Conclusions**: These findings underscore the role of sleep duration and regularity in shaping body composition and cardiometabolic health, supporting its relevance as a modifiable public health priority.

## 1. Introduction

Sleep is a fundamental physiological process essential for sustaining life and supporting human function. In recent years, the prevalence of sleep-related problems has increased substantially worldwide, with an estimated 10–30% of adults experiencing sleep disturbances or poor sleep quality [1,2]. This trend has raised growing concerns among health professionals, as it represents an emerging and significant public health issue.

A substantial body of evidence indicates that insufficient or irregular sleep patterns—shaped by various socio-demographic and lifestyle factors—are associated with a range of adverse health outcomes, particularly regarding cardiometabolic risk, through a complex network of physiological mechanisms [3,4,5]. Short or fragmented sleep has been linked to increased levels of ghrelin and decreased levels of leptin—two hormones involved in appetite regulation—potentially leading to increased food intake and weight gain [6]. Additionally, sleep deprivation impairs insulin sensitivity and glucose tolerance, increasing the likelihood of insulin resistance and, over time, the development of type 2 diabetes [7]. Chronic disturbances in sleep have also been associated with elevated blood pressure, systemic inflammation, and endothelial dysfunction, all of which are key contributors to cardiovascular disease [8,9,10]. Beyond these physiological effects, disrupted sleep also appears to influence health-related behaviors, particularly those related to diet. For example, insufficient or poor-quality sleep has been associated with a preference for calorie-dense foods rich in sugar and fat and irregular eating patterns [11]. Conversely, certain dietary components, such as fiber, antioxidants, and vitamins, have been hypothesized to promote better sleep quality, although empirical evidence remains mixed [12,13].

Despite growing interest in these associations, much of the current literature relies on self-reported sleep measures and focuses on sleep duration in isolation, overlooking the potential combined effects of multiple objectively monitored sleep dimensions. Within the realm of cardiometabolic markers of health, characteristics such as sleep efficiency—the proportion of time spent asleep relative to time spent in bed—and sleep regularity, which reflects the consistency of sleep and wake times across days, remain largely underexplored. To address these gaps, the present study investigates the associations between key objective sleep parameters—namely, duration, efficiency, and regularity—and cardiometabolic markers, body composition, and dietary patterns in a sample of healthy adults, through 7-day actigraphy-based sleep assessments, a detailed food diary, and biochemical profile analyses.

## 2. Materials and Methods

### 2.1. Study Design and Participants

This study reports baseline data collected from a dietary intervention trial conducted at the Unit of Clinical Nutrition of Careggi University Hospital in Florence, Italy, for which the protocol has already been published elsewhere [14]. The recruitment phase took place between October 2023 and December 2024. Adults aged 18 to 65 years, of both sexes, with a body mass index (BMI) between 18.5 and 24.9 kg/m^2^ were eligible for inclusion in the study. Exclusion criteria comprised individuals engaged in night shift work, those planning long-distance travel, or those with irregular sleep schedules. Additionally, participants using medications known to affect sleep or metabolism, or those with ongoing medical conditions requiring dietary management (such as recent myocardial infarction, chronic liver disease, or diabetes) were excluded. Further exclusion criteria included pregnancy, intention to become pregnant within the next 12 months, breastfeeding, and current or recent (within the last three months) use of supplements or antibiotics. The inclusion and exclusion criteria applied in this study were those defined in the main clinical trial [14]. Informed consent was obtained from all participants after a detailed explanation of the study procedures.

Ethical approval was obtained from the Ethics Committee (spe123.23, date 26 September 2023) of the Tuscany Region, Careggi University Hospital, Florence. The study was conducted in accordance with the principles of the Declaration of Helsinki and applicable data protection regulations.

### 2.2. Data Collection

During the initial enrollment visit, participants were informed about the study objectives and data collection procedures. Upon providing informed voluntary consent, each participant received an actigraph to be worn continuously for seven consecutive days, a 3-day food diary and the Medi-Lite questionnaire to completed [15]. Physical activity was also assessed using the long version of the International Physical Activity Questionnaire (IPAQ) [16]. After one week, participants attended a scheduled morning visit, held between 7:30 and 10:30 a.m., following a 12 h overnight fast. During this visit, the completed actigraph, food diary, Medi-Lite and IPAQs were collected, and participants underwent body composition assessment and venous blood sampling.

#### 2.2.1. Sleep Analysis

Sleep–wake cycle analysis was performed using actigraphy, a non-invasive method that involves wearing a wrist-worn actigraph continuously for seven consecutive days. In this study, sleep was monitored using the Fitbit Alta HR device. While consumer devices are generally less precise than research-grade actigraphy, validation studies have demonstrated acceptable reliability of the Fitbit Alta HR for measuring sleep parameters in free-living conditions. For example, Kawasaki et al. reported strong correlations between Fitbit Alta HR and electroencephalogram-derived total sleep time in healthy adults (R = 0.83), and Moreno-Pino et al. found acceptable agreement with polysomnography for sleep duration [17,18]. The Fitbit Alta HR was chosen for its ease of use, high participant adherence, and capability for continuous long-term monitoring outside laboratory settings, which is often challenging with research-grade actigraphy.

Participants were instructed to wear the actigraph on their non-dominant hand and not to remove it until the visit with the research team. Data recorded by the actigraph were subsequently downloaded and processed using the DORMI—Sleep Analysis Evolution software, developed by Sleepacta (Pisa, Italy). DORMI is a certified software, included in the register of Class I medical devices of the Italian Ministry of Health since 15 November 2018. It analyses movement data and specific physiological parameters captured by the actigraph to generate a comprehensive set of sleep metrics. The primary variables extracted from the actigraphy data included the Total Sleep Time (TST), representing the total sleep duration in hours and minutes, the Sleep Efficiency (SE), calculated as the percentage of time spent asleep relative to time in bed (in %), and Sleep Regularity Index (SRI) a metric that quantifies the consistency of an individual’s sleep–wake patterns across days. These metrics are commonly used in studies employing wearable devices for sleep assessment [19,20,21,22].

#### 2.2.2. Anthropometric Parameters and Body Composition

Height was measured using a stadiometer, and weight was recorded with a professional digital scale (TANITA, model TBF-410, Tokyo, Japan) with a precision of 0.1 kg before the assessment. BMI was calculated as weight (kg)/height (m^2^). Body composition was assessed using a bioelectrical impedance analyzer (Akern, model SE 101, Pisa, Italy). Prior to the assessment, participants were instructed to follow standardized pre-test conditions, including abstaining from vigorous physical activity for at least 24 h and from food and fluid intake for 4 h. During the body composition assessment, participants were positioned supine with their limbs slightly abducted from the body—arms approximately 30° away from the trunk and legs separated by about 45°. Four disposable electrodes were placed according to the standard protocol: two on the dorsal surfaces of the dominant hand (wrist and metacarpal region) and two on the dominant foot (ankle and metatarsal region). The device applied a low-level electrical current and measured resistance and reactance values. These data, together with participant information including sex, age, weight, and height, were entered into the Bodygram Plus software, which used proprietary algorithms to estimate body composition. The derived variables included fat mass, fat-free mass, total body water, intracellular water, extracellular water, skeletal muscle mass, body cellular mass, and phase angle. Phase angle reflects cell membrane integrity and overall cellular health, while body cell mass represents the metabolically active, fat-free portion of the body.

The measurement typically lasted less than 5 min and was conducted in a controlled environment, with participants wearing light clothing and no metallic accessories to avoid interference.

#### 2.2.3. Biochemical Profile

During the visit, a blood sample from the participants was collected. Blood samples were centrifuged at 3000 rpm for 15 min to separate the serum, which was then aliquoted and stored at −20 °C until analysis. Biochemical analyses were conducted according to standardized laboratory protocols. The following parameters were assessed in all participants: complete blood count; glycemic profile (fasting glucose and glycated hemoglobin), homocysteine levels, lipid profile (total cholesterol, LDL cholesterol, HDL cholesterol, triglycerides), liver function markers [aspartate aminotransferase (AST), alanine aminotransferase (ALT), and gamma-glutamyl transferase (γGT)], renal function indicators (serum creatinine, urea, uric acid), mineral profile (sodium, potassium, magnesium, calcium), iron metabolism parameters (serum iron, ferritin) and vitamin levels (vitamin B12 and folic acid).

#### 2.2.4. Dietary Habits

Nutritional intake and habitual mealtimes of the participants were assessed using a 3-day standardized weighted dietary record (WDR). During the distribution of the materials, the research team provided detailed guidance on how to complete the WDR as accurately and precisely as possible. Participants were asked to record all foods and beverages consumed, including portion sizes in grams or milliliters, preparation methods and time of consumption. They were also instructed to complete on two weekdays and one weekend day to capture habitual dietary patterns. Trained personnel entered the data into the Metadieta 4.6 software application (Me.Te.Da., San Benedetto del Tronto, Italy), thoroughly checked for errors, and analyzed it to determine energy and nutrient intakes, including macronutrients (carbohydrates, fat, protein) and micronutrients. The temporal pattern of food consumption was analyzed by calculating the feeding window, the midpoint of energy intake (EI), and the time of last meal. The feeding window is the time between the first and last meal of the day, while the midpoint of EI is the midpoint between the time of the first and last meal of the day. These variables were selected based on studies linking them to negative health outcomes, such as cardiovascular risk and obesity [23,24,25].

Adherence to the Mediterranean Diet (MD) was assessed using the validated Medi-Lite questionnaire [15]. The Medi-Lite adherence score includes nine items assessing daily intake of fruit, vegetables, cereals, meat and meat products, dairy products, alcohol, and olive oil, as well as weekly intake of legumes and fish. Each food group in the score is classified into three levels of consumption, scoring between 0 and 2 points. The total score ranges from 0 (low adherence) to 18 (high adherence).

### 2.3. Statistical Analysis

Statistical analyses were conducted using the PASW Statistics 27.0 software for Macintosh (SPSS Inc., Chicago, IL, USA). Continuous variables were expressed as means ± standard deviations (SD), while categorical variables were presented as frequencies and percentages (%). Group differences for continuous variables were assessed using the Mann–Whitney U test, whereas the Chi-square test was applied for categorical variables. Correlations between variables were examined using Spearman’s rank correlation coefficient. Furthermore, for variables showing a significant correlation, the parameter of interest was analyzed using a multivariable linear regression model adjusted for potential confounders, including age, sex, physical activity, smoking habits, and total energy intake. Results are reported as unstandardized β coefficients with 95% confidence intervals (CI). For all tests, a *p*-value < 0.05 was considered statistically significant.

Actigraphy-derived sleep variables were categorized based on thresholds commonly used in the literature to ensure comparability and interpretability. TST was dichotomized as <7 h versus ≥7 h per night, reflecting recommended sleep duration for adults [20]. SRI was divided into quintiles, with comparisons focused on the lowest (1st quintile; ≤41) and highest (5th quintile; ≥80) groups, as performed in a previous study [21]. SE was classified as <85% versus ≥85%. This cut-off was used to demonstrate that participants have objectively measured disturbed sleep in addition to meeting the DSM-5 criteria for insomnia [22].

## 3. Results

### 3.1. Characteristics of the Study Sample

Table 1 provides a summary of the characteristics of the study participants, who included a total of 70 subjects, predominantly females (74.3%). The mean age of the sample was 28.3 ± 10.1 years, and the mean BMI was 21.9 ± 2.1 kg/m^2^. Most were unmarried (80%), without children (85.7%), and held a university degree (55.8%). The analysis of the IPAQ revealed that only 7.1% of the participants were sedentary, compared to the majority who were sufficiently active (54.3%) or active (38.6%). Furthermore, 25.7% of the sample participants stated that they were smokers.

Sleep analysis showed that participants had a good sleep pattern with a mean TST of 7.4 ± 1.1 h, a SE of 84.9 ± 6.9%, and an SRI of 62.2 ± 19.9. The only notable difference observed was that participants in the lowest quintile of SRI (1st) were significantly younger than those in the highest quintile (5th).

### 3.2. Anthropometric Parameters and Body Composition

**Figure 1:**
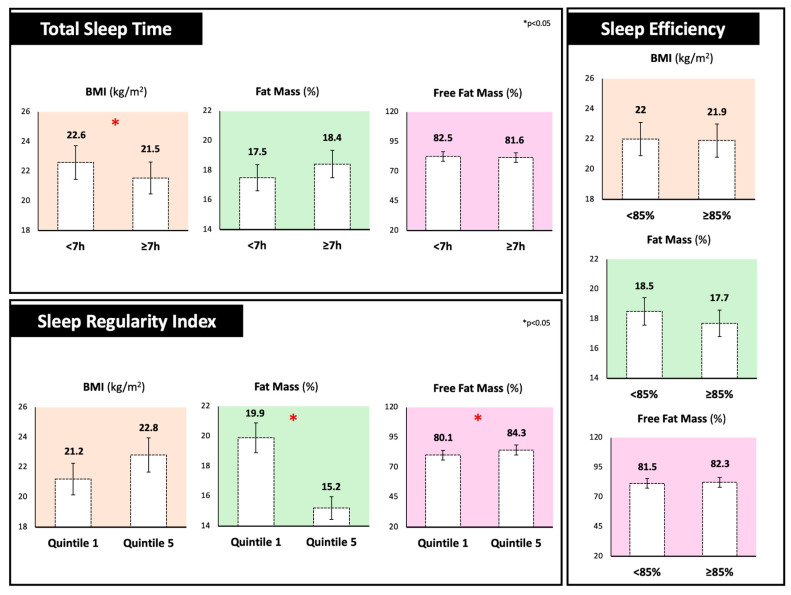
Body mass index, fat mass, and fat-free mass percentages of participants stratified by different sleep components. Total Sleep Time was dichotomized as <7 h versus ≥7 h per night; Sleep Regularity Index was divided into quintiles (1st quintile; ≤41) and highest (5th quintile; ≥80) groups and Sleep Efficiency was classified as <85% versus ≥85%.

Figure 1 shows anthropometric and body composition analysis stratified by different sleep components. Individuals sleeping less than 7 h per night exhibited significantly higher BMI values compared to those sleeping 7 h or more (22.6 ± 2.1 vs. 21.5 ± 2.0 kg/m^2^, *p* < 0.05). Notably, the SRI was also associated with body composition parameters, with participants in the lowest SRI quintile (1st) showing a significantly higher percentage of fat mass (+4.7%) and a lower percentage of fat-free mass (−4.7%) compared to those in the highest quintile (5th). In contrast, no significant differences were observed in relation to SE values. For the other body composition parameters, no significant differences emerged.

A slight, but significant, inverse correlation was observed between BMI and TST (R= −0.26, *p* = 0.02), suggesting that higher BMI is associated with shorter sleep duration. However, in the adjusted linear regression model, controlling for age, sex, physical activity, smoking, and total energy intake, BMI was not significantly associated with TST (β= −0.20, 95% CI: −0.63 to 0.23; *p* = 0.35).

### 3.3. Biochemical Profile

Biochemical profile analysis is reported in Table 2. Interestingly, participants in the lowest SRI quintile (1st) showed a significantly less favorable lipid profile, characterized by higher total cholesterol (183.9 ± 20.9 vs. 155.0 ± 26.8 mg/dL; *p* = 0.008) and lower HDL cholesterol levels (65.1 ± 14.8 vs. 78.6 ± 15.8 mg/dL; *p* = 0.03) compared to the participants in the highest SRI quintile (5th). This group had also significantly lower folate concentrations (3.6 ± 1.6 vs. 5.6 ± 2.5 ng/mL; *p* = 0.02). Similarly, sleeping less than 7 h per night exhibited significantly higher homocysteine levels compared to those sleeping 7 h or more (11.4 ± 2.3 vs. 10.4 ± 3.3 μmol/L; *p* = 0.03). In contrast, no significant findings were observed in relation to SE.

A slight, but significant, negative association was found between total cholesterol levels and SRI (R= −0.28, *p* = 0.01), indicating that increased cholesterol levels are linked to poorer sleep regularity. However, in the adjusted linear regression model cholesterol was not significantly associated with SRI (β= −0.17, 95% CI: −0.21 to 0.55; *p* = 0.37).

### 3.4. Dietary Habits

Analysis of the 3-day food diaries revealed no significant differences in total EI, macronutrient distribution, or adherence to the MD across the three components of sleep analyzed, as shown in Table 3. However, participants reporting less than 7 h of sleep per night had a significantly higher intake of energy from animal protein (53.9 ± 16.2% vs. 40.6 ± 10.4% of energy; *p* = 0.04) and sodium (1947.8 ± 929.2 mg vs. 1478.6 ± 666.9 mg; *p* = 0.03) compared to those sleeping more than 7 h. Here, the percentage of energy from animal protein indicates the share of total daily energy derived from animal-based protein sources. Additionally, individuals with SE < 85% consumed significantly less vitamin A (915.5 ± 517.9 µg vs. 1245.8 ± 707.2 µg; *p* = 0.04) than those with higher efficiency.

Regarding temporal eating behavior, no major differences were found in the timing of meals or in the overall eating window and energy midpoint across sleep parameters. The only significant variation was in the caloric contribution of the mid-afternoon snack, which was lower among participants sleeping less than 7 h (3.5 ± 4.5% vs. 10.5 ± 7.0%; *p* = 0.01).

## 4. Discussion

The present study investigated the relationship between objectively measured sleep parameters and markers of body composition, cardiometabolic health, and dietary intake in a sample of healthy, normal-weight adults. The main findings highlight that individuals in the lowest quintile of SRI showed a significantly higher fat mass percentage, increased total plasma cholesterol, and lower HDL cholesterol and folate levels. Similarly, participants who slept less than 7 h per night had significantly higher BMI and homocysteine levels. Moreover, analysis of individual dietary components showed that shorter TST correlated with higher intake of animal protein and sodium. Contrary to expectations, a lower SE was not associated with significant differences in most metabolic or dietary parameters, except for a significantly lower intake of vitamin A.

Our findings regarding sleep regularity are consistent with prior evidence emphasizing the relevance of circadian alignment in metabolic regulation [26,27]. In our sample, individuals with the most irregular sleep–wake patterns (i.e., lowest SRI quintile) showed a less favorable cardiometabolic profile compared to those with more consistent schedules, reporting higher fat mass percentage, increased total plasma cholesterol, and lower HDL cholesterol. Irregular sleep–wake patterns may disrupt the synchrony between the central circadian pacemaker located in the suprachiasmatic nucleus and peripheral clocks in metabolic tissues such as adipose tissue, liver, and skeletal muscle [28]. In particular, animal studies suggest that circadian misalignment induced by irregular sleep timing can disrupt the hepatic expression of key regulators of cholesterol biosynthesis, lipoprotein assembly, and bile acid metabolism, including HMG-CoA reductase, SREBP-1c, and ABCA1 [29,30,31]. Such alterations may lead to dysregulation of lipid profiles and contribute to an unfavorable cardiometabolic risk profile. Taken together, these findings point toward a shared mechanism through which sleep irregularity may negatively impact both body composition and lipid metabolism via circadian disruption. Further research is warranted to determine whether similar processes occur in humans and to elucidate the full spectrum of metabolic consequences associated with irregular sleep patterns in everyday life. It is also important to consider that the directionality of the observed associations may be bidirectional. Higher fat mass or altered lipid profiles could impair sleep regularity. Excess adiposity has been associated with chronic low-grade inflammation and hormonal dysregulation, including altered leptin signaling, which may negatively affect sleep–wake stability [32].

In addition to lipid profile, individuals with poorer sleep regularity showed a significantly lower serum folate concentration. This finding aligns with recent results from Tu and colleagues, who, in a sample of 20,200 American adults, reported that individuals with suboptimal folate status were more likely to experience self-reported sleep disturbances, including irregular sleep–wake rhythms [33]. The authors proposed that folate may influence sleep through its role in essential metabolic pathways, particularly one-carbon metabolism [33]. This pathway is central to DNA synthesis and methylation, processes that in turn regulate gene expression—including the expression of genes involved in circadian rhythms and the synthesis of key neurotransmitters such as serotonin and melatonin [34,35]. Nonetheless, the relationship between serum folate levels and sleep regularity should be interpreted with caution, as several other studies have reported no association between these variables [36,37].

From the analysis of sleep duration, we observed that participants sleeping less than 7 h per night exhibited significantly higher BMI values, in line with previous observations in healthy-weight populations [38,39]. Several physiological mechanisms may underlie this relationship, including reduced leptin and elevated ghrelin levels, decreased insulin sensitivity, and increased evening cortisol secretion—all of which contribute to impaired metabolic homeostasis [40,41].

Interestingly, analysis of individual dietary components showed that shorter TST correlated with higher intake of animal protein and sodium. Similar findings were reported by Wirth and colleagues, who noted that plant-based protein intake was linked to better sleep quality, possibly due to the higher carbohydrate content of plant sources, which may promote a healthier sleep pattern [42]. The association with sodium may reflect its potential role in increasing nocturnal urination, thereby disrupting sleep continuity [43].

Furthermore, participants with shorter sleep duration showed significantly higher levels of homocysteine, an independent and well-recognized risk factor for endothelial dysfunction, atherogenesis and cardiovascular disease [44]. To date, most of the available literature has focused on investigating homocysteine levels in relation to obstructive sleep apnea [45]. Although the absolute difference in homocysteine levels observed in our sample is small, it is consistent with the limited evidence available in populations with normal sleep patterns. This includes evidence from several studies based on the US National Health and Nutrition Examination Survey (NHANES), which demonstrated that individuals sleeping five hours or less had significantly higher homocysteine levels compared to those sleeping 7 h [46,47]. While the mechanisms linking short TST to elevated homocysteine remain to be fully elucidated, some studies indicate that sleep deprivation impairs homocysteine metabolism via disrupted redox balance and methylation pathways [48,49]. In humans, even a single night of sleep loss reduces plasma glutathione and ATP levels, alters DNA methylation, and increases homocysteine [49]. If such alterations persist over time, even modest elevations could contribute to long-term cardiometabolic risk. Longitudinal studies are needed to determine the clinical relevance of these findings.

Regarding SE, no significant differences were found in any cardiometabolic parameters. The only exception was dietary vitamin A intake, which was higher among individuals with greater SE. While the cross-sectional design of the present study precludes causal inference, this result may be biologically plausible given the known role of vitamin A in circadian regulation and sleep physiology. Specifically, vitamin A contributes to the synthesis of retinoic acid, a metabolite that modulates gene expression via nuclear receptors [50]. Retinoic acid signaling plays a key role in maintaining the rhythmic expression of central clock genes such as PER1 and BMAL1, which are crucial for proper sleep–wake cycle regulation [50].

The findings of this study should be interpreted with caution, as several limitations must be acknowledged. First, the cross-sectional design prevents the assessment of causal relationships between variables. Second, the sample size of this study is skewed toward female participants and was determined based on the primary endpoint of the main clinical trial [14]. The baseline analyses examining associations between sleep, metabolic, and dietary variables are exploratory in nature. Moreover, the exclusion of individuals with overweight/obesity and chronic diseases, combined with the recruitment of mostly young, physically active, and highly educated subjects, limits the applicability of these findings to populations typically at highest cardiometabolic risk and restricts the generalizability of the results to the broader population. In addition, self-reported dietary data may be subject to recall bias. Nevertheless, the study employed highly specific methods to assess the outcomes of interest. Sleep patterns were objectively monitored using actigraphy over a continuous 7-day period, providing a reliable and valid measure of sleep behavior. Dietary intake was assessed with a structured and validated 3-day food diary, allowing for a detailed evaluation of nutrient intake. These methodological strengths contribute to the robustness of the findings and support the relevance of further investigation in this area.

## 5. Conclusions

In conclusion, this study adds to the current literature by describing how different sleep parameters may relate to indicators of body composition, cardiometabolic health, and diet in healthy adults. Irregular sleep patterns were observed in those with greater fat mass, less favorable lipid profiles, and lower serum folate, while shorter sleep duration was observed in individuals with higher BMI and homocysteine levels. These initial findings could suggest that maintaining regular sleep patterns may support better metabolic and cardiovascular health. Given the rising prevalence of poor and irregular sleep habits in modern societies, further research is urgently needed to explore underlying biological mechanisms and evaluate whether promoting healthier sleep duration and regularity may serve as effective, non-pharmacological strategies to enhance cardiometabolic health and overall well-being.

## Figures and Tables

**Table 1:** Demographic and lifestyle characteristics of participants in the total sample and according to the different components of sleep.

	All (*n* = 70)	Total Sleep		*p*-Value ^a^	Sleep Regularity		*p*-Value ^b^	Sleep Efficiency		*p*-Value ^c^
		<7 h (*n* = 27)	≥7 h (*n* = 43)		1st Quintile (*n* = 14)	5th Quintile (*n* = 14)		<85% (*n* = 32)	≥85% (*n* = 38)	
**Sex**										
Female, *n* (%)	52 (74.3)	17 (63)	35 (81.4)	0.08	11 (78.6)	11 (78.6)	1.0	26 (81.3)	26 (68.4)	0.22
**Age** (years) °	28.3 ± 10.1	29.8 ± 11.8	27.4 ± 8.9	0.63	23.1 ± 2.7	36.1 ± 12.3	<0.001 *	30.7 ± 12.1	26.4 ± 7.7	0.38
**Marital status**										
Unmarried/single, *n* (%)	56 (80)	21 (77.8)	35 (81.4)	0.52	11 (78.6)	9 (64.2)	0.37	25 (78.2)	31 (81.5)	0.68
Married/partner, *n* (%)	14 (20)	6 (22.2)	8 (18.6)		3 (21.4)	5 (35.7)		7 (21.8)	7 (18.5)	
**Having children**, *n* (%)	10 (14.3)	5 (18.5)	5 (11.6)	0.42	1 (7.1)	4 (28.6)	0.13	6 (18.8)	4 (10.5)	0.32
**Education**										
Elementary/Middle school, *n* (%)	4 (5.7)	2 (7.4)	2 (4.6)	0.48	1 (7.1)	1 (7.1)	0.54	2 (6.3)	2 (5.2)	0.55
High school, *n* (%)	27 (38.6)	12 (44.4)	15 (34.9)		7 (50)	6 (42.9)		14 (43.8)	13 (34.2)	
University, *n* (%)	39 (55.8)	13 (48.1)	26 (60.5)		6 (42.9)	7 (50)		16 (50)	23 (60.5)	
**Physical activity**										
Sedentary, *n* (%)	5 (7.1)	1 (3.7)	4 (9.3)	0.55	-	1 (7.1)	0.24	3 (9.4)	2 (5.3)	0.69
Sufficiently active, *n* (%)	38 (54.3)	14 (51.9)	24 (55.8)		9 (64.3)	5 (35.7)		18 (56.3)	20 (52.6)	
Active, *n (%)*	27 (38.6)	12 (44.4)	15 (34.9)		5 (35.7)	8 (57.1)		11 (34.4)	16 (42.1)	
**Smokers**, *n* (%)	18 (25.7)	10 (37)	8 (18.6)	0.11	4 (28.6)	5 (35.7)	0.90	10 (31.3)	8 (21.1)	0.39

* *p* < 0.05; ° Mean ± Standard Deviation. *p*-value ^a^: comparison of total sleep < 7 h vs. ≥7 h; *p*-value ^b^: comparison of sleep regularity 1st quintile vs. 5th quintile; *p*-value ^c^: comparison of sleep efficiency < 85% vs. ≥85%.

**Table 2:** Biochemical profile of participants according to the different components of sleep.

	Total Sleep		*p*-Value ^a^	Sleep Regularity		*p*-Value ^b^	Sleep Efficiency		*p*-Value ^c^
	<7 h (*n* = 27)	≥7 h (*n* = 43)		1st Quintile (*n* = 14)	5th Quintile (*n* = 14)		<85% (*n* = 32)	≥85% (*n* = 38)	
**Homocysteine** (μmol/L)	11.4 ± 2.3	10.4 ± 3.3	0.03 *	10.8 ± 3.0	10.2 ± 2.8	0.66	11.1 ± 3.1	10.6 ± 2.9	0.65
**Glucose** (mg/dL)	81.0 ± 7.8	78.8 ± 8.6	0.31	80.5 ± 8.0	79.1 ± 11.6	0.98	78.9 ± 10.0	80.3 ± 7.1	0.76
**HbA1c** (mmol/mol)	33.1 ± 3.8	32.2 ± 3.3	0.44	31.4 ± 3.2	33.7 ± 4.0	0.17	32.7 ± 4.5	32.4 ± 2.5	0.95
**Total cholesterol** (mg/dL)	169.1 ± 36.2	166.0 ± 26.9	0.63	183.9 ± 20.9	155.0 ± 26.8	0.008 *	168.9 ± 36.5	165.8 ± 25.0	0.80
**LDL-cholesterol** (mg/dL)	89.1 ± 32.8	85.4 ± 23.3	0.81	76.2 ± 22.7	91.1 ± 21.3	0.12	87.3 ± 31.6	86.5 ± 23.3	0.87
**HDL-cholesterol** (mg/dL)	65.4 ± 16.4	65.9 ± 13.6	0.71	65.1 ± 14.8	78.6 ± 15.8	0.03 *	66.5 ± 14.1	65.1 ± 15.2	0.56
**Triglycerides** (mg/dL)	72.6 ± 27.0	73.0 ± 26.5	0.79	68.7 ± 34.1	71.3 ± 16.0	0.37	75.2 ± 28.3	71.0 ± 25.2	0.62
**AST** (U/L)	19.5 ± 6.4	22.6 ± 13.9	0.68	22.4 ± 8.5	24.6 ± 11.3	0.66	21.6 ± 10.1	21.2 ± 12.9	0.49
**ALT** (U/L)	18.3 ± 14.0	18.2 ± 14.5	0.93	18.0 ± 9.5	25.4 ± 25.1	0.73	18.4 ± 14.6	18.1 ± 14.0	0.66
**Gamma-GT** (U/L)	14.8 ± 5.5	15.4 ± 8.4	0.82	14.9 ± 6.1	17.3 ± 10.9	0.51	16.2 ± 8.9	14.3 ± 5.9	0.43
**Creatinine** (mg/dL)	0.8 ± 0.1	0.8 ± 0.1	0.51	0.8 ± 0.2	0.8 ± 0.1	0.54	0.8 ± 0.1	0.8 ± 0.2	0.95
**Uric acid** (mg/dL)	4.4 ± 1.1	4.2 ± 0.9	0.24	4.5 ± 1.3	4.2 ± 1.2	0.76	4.3 ± 1.0	4.2 ± 1.0	0.75
**Folate** (ng/mL)	4.6 ± 3.3	6.0 ± 5.5	0.07	4.0 ± 1.6	5.6 ± 2.5	0.02 *	5.2 ± 4.1	5.7 ± 5.4	0.38
**Vitamin B12** (pg/mL)	414.6 ± 442.5	442.5 ± 163.4	0.74	411.8 ± 168.7	486.1 ± 152.3	0.16	422.2 ± 146.9	439.8 ± 144.7	0.37
**Ferritin** (ng/mL)	77.9 ± 63.3	57.4 ± 52.5	0.15	45.1 ± 25.7	70.6 ± 68.0	0.63	56.0 ± 41.8	73.1 ± 67.4	0.46
**Iron** (μg/dL)	92.1 ± 40.5	100.3 ± 41.1	0.55	97.4 ± 45.5	89.2 ± 35.7	0.45	98.3 ± 37.4	96.1 ± 43.9	0.50
**Sodium** (mEq/dL)	140.4 ± 1.6	140.4 ± 1.5	0.91	140.3 ± 1.6	140.7 ± 1.5	0.51	140.6 ± 1.3	140.3 ± 1.6	0.49
**Potassium** (mEq/dL)	4.3 ± 0.3	4.3 ± 0.3	0.62	4.3 ± 0.3	4.4 ± 0.3	0.42	4.3 ± 0.3	4.3 ± 0.3	0.85
**Magnesium** (mg/dL)	2.0 ± 0.1	2.1 ± 0.5	0.06	2.0 ± 0.1	2.3 ± 0.8	0.15	2.1 ± 0.5	2.0 ± 0.1	0.26

ALT: Alanine aminotransferases; AST: Aspartate aminotransferases; GT: Glutamyl Transferase; HbA1c: Glycated hemoglobin; HDL: High Density Lipoprotein; LDL: Low Density Lipoprotein. *p*-value ^a^: comparison of total sleep < 7 h vs. ≥7 h; *p*-value ^b^: comparison of sleep regularity 1st quintile vs. 5th quintile; *p*-value ^c^: comparison of sleep efficiency < 85% vs. ≥85%. * *p* < 0.05.

**Table 3:** Dietary habits of participants according to the different components of sleep.

	Total Sleep		*p*-Value ^a^	Sleep Regularity		*p*-Value ^b^	Sleep Efficiency		*p*-Value ^c^
	<7 h (*n* = 27)	>7 h (*n* = 43)		1st Quintile (*n* = 14)	5th Quintile (*n* = 14)		<85% (*n* = 32)	>85% (*n* = 38)	
**DIETARY INTAKE**									
Total energy, kcal/day	1992.9 ± 464.5	1835.1 ± 457.1	0.21	1920.3 ± 551.9	1818.6 ± 327.1	0.80	1840.7 ± 494.3	1942.5 ± 435.4	0.17
Carbohydrates, % of energy	46.0 ± 7.8	45.9 ± 8.4	0.80	45.6 ± 7.0	43.0 ± 8.0	0.19	47.7 ± 7.3	44.4 ± 8.6	0.18
Protein, % of energy	16.6 ± 2.4	16.6 ± 3.3	0.80	18.3 ± 3.6	17.5 ± 3.4	0.48	16.8 ± 3.2	16.4 ± 2.8	0.70
Animal proteins, % of energy	53.6 ± 16.2	45.5 ± 15.4	0.04 *	44.4 ± 14.3	52.2 ± 19.3	0.10	47.9 ± 13.7	49.2 ± 18.0	0.45
Plant-based proteins, % of energy	34.1 ± 13.8	37.6 ± 13.9	0.19	39.7 ± 12.5	35.7 ± 16.5	0.16	37.1 ± 11.7	35.6 ± 15.6	0.22
Total fat, % of energy	37.2 ± 5.8	36.3 ± 6.0	0.76	35.6 ± 6.0	39.5 ± 6.3	0.16	35.3 ± 5.9	37.8 ± 5.7	0.07
Total cholesterol, mg	225.3 ± 92.5	190.1 ± 96.0	0.18	212.3 ± 116.2	217.2 ± 116.3	0.73	195.4 ± 90.5	210.6 ± 100.4	0.47
Saturated fat, % of energy	22.0 ± 7.1	20.3 ± 6.9	0.44	19.4 ± 5.8	19.7 ± 4.5	0.94	19.9 ± 6.3	21.8 ± 7.5	0.45
Fiber, g	20.9 ± 9.5	21.4 ± 9.0	0.57	24.2 ± 10.9	22.4 ± 8.7	0.45	22.4 ± 10.5	20.2 ± 7.8	0.58
Calcium, mg	614.7 ± 263.7	593.8 ± 193.0	0.81	633.8 ± 206.4	573.6 ± 200.0	0.26	574.7 ± 206.1	624.7 ± 233.7	0.31
Potassium, mg	2506.3 ± 697.5	2518.2 ± 939.2	0.70	2752.5 ± 970.7	2548.2 ± 396.2	0.51	2530.6 ± 941.4	2499.3 ± 774.7	0.75
Sodium, mg	1947.8 ± 929.2	1478.6 ± 662.1	0.03 *	1482.4 ± 640.4	1544.3 ± 719.8	0.83	1587.1 ± 945.4	1720.6 ± 667.7	0.21
Iron, mg	12.2 ± 4.5	10.4 ± 4.0	0.10	12.2 ± 4.9	12.3 ± 4.3	0.63	11.2 ± 5.6	11.1 ± 4.0	0.81
Zinc, mg	9.5 ± 3.0	8.7 ± 3.0	0.21	9.2 ± 3.4	9.3 ± 3.1	0.73	8.8 ± 3.1	9.1 ± 3.0	0.38
Copper, mg	0.9 ± 0.4	0.9 ± 0.4	0.97	1.0 ± 0.4	0.8 ± 0.3	0.12	0.9 ± 0.4	0.9 ± 0.4	0.96
Thiamine, mg	1.1 ± 0.4	0.9 ± 0.3	0.15	1.0 ± 0.4	1.0 ± 0.3	0.70	1.0 ± 0.4	1.0 ± 0.3	0.93
Riboflavin, mg	1.5 ± 0.4	1.4 ± 0.4	0.37	1.5 ± 0.6	1.4 ± 0.3	0.63	1.4 ± 0.4	1.4 ± 0.4	0.96
Vitamin A, mcg	1051.3 ± 607.4	1122.1 ± 673.3	0.70	1353.6 ± 733.2	1166.7 ± 576.9	0.42	915.5 ± 517.9	1245.8 ± 707.2	0.04 *
Vitamin C, mcg	92.4 ± 58.7	93.9 ± 55.7	0.70	97.5 ± 60.0	101.1 ± 50.8	0.87	86.0 ± 45.7	99.4 ± 64.2	0.56
**TEMPORAL PATTERN**									
Caloric distribution, %									
Breakfast	14.5 ± 7.1	13.3 ± 6.1	0.64	12.1 ± 7.2	13.3 ± 5.2	0.94	13.9 ± 7.1	14.0 ± 6.0	0.71
Mid morning snack	3.8 ± 4.1	3.8 ± 5.1	0.75	5.4 ± 6.7	4.0 ± 5.0	0.83	3.8 ± 4.3	3.9 ± 5.0	0.77
Lunch	34.3 ± 7.4	36.5 ± 8.9	0.24	36.5 ± 8.0	37.7 ± 8.9	0.60	34.8 ± 9.2	36.4 ± 7.6	0.49
Mid afternoon snack	4.6 ± 4.5	8.2 ± 5.1	0.002 *	10.0 ± 7.2	7.2 ± 4.3	0.28	7.0 ± 5.5	6.6 ± 5.0	0.99
Dinner	39.9 ± 8.5	39.0 ± 8.7	0.73	39.2 ± 10.4	38.4 ± 9.2	0.91	38.5 ± 8.0	40.1 ± 9.0	0.70
Evening snack	1.5 ± 3.9	0.7 ± 1.7	0.99	0.6 ± 1.7	0.5 ± 1.4	0.98	1.0 ± 2.4	1.0 ± 3.1	0.91
Eating window, hh:mm	13:15 ± 1:37	12:20 ± 1:35	0.06	12:39 ± 1:40	12:13 ± 1:15	0.37	12:47 ± 1:48	12:37 ± 1:31	0.84
Eating midpoint of energy, hh:mm	02:26 ± 0:54 p.m.	02:18 ± 1:51 p.m.	0.34	02:30 ± 0:49 p.m.	02:23 ± 0:56 p.m.	0.57	02:26 ± 0:44 p.m.	02:17 ± 2:01 p.m.	0.79
Last feeding occasion, hh:mm	07:16 ± 4:17 p.m.	08:31 ± 1:37 p.m.	0.31	08:47 ± 1:01 p.m.	07:38 ± 2:18 p.m.	0.12	08:27 ± 1:50 p.m.	07:42 ± 3:40 p.m.	0.76
**MD ADHERENCE**									
Medi-Lite total score	10.3 ± 2.5	10.9 ± 2.4	0.27	11.9 ± 2.5	10.6 ± 2.6	0.16	11.0 ± 2.5	10.4 ± 2.4	0.33

MD: Mediterranean diet; * *p* < 0.05. *p*-value ^a^: comparison of total sleep < 7 h vs. ≥7 h; *p*-value ^b^: comparison of sleep regularity 1st quintile vs. 5th quintile; *p*-value ^c^: comparison of sleep efficiency < 85% vs. ≥85%.

## Data Availability

The data that support the findings of this study are available from the corresponding authors upon reasonable request due to ethical considerations, as the main clinical trial is still ongoing.

## Abbreviations

The following abbreviations are used in this manuscript:

ABCA1	ATP-Binding Cassette Transporter A1
ALT	Alanine Aminotransferase
AST	Aspartate Aminotransferase
ATP	Adenosine Triphosphate
BMI	Body Mass Index
EI	Energy Intake
HDL	High-Density Lipoprotein
HMG-CoA	3-Hydroxy-3-Methylglutaryl-Coenzyme A reductase
IPAQ	International Physical Activity Questionnaire
LDL	Low-Density Lipoprotein
MD	Mediterranean Diet
NHANES	National Health and Nutrition Examination Survey
SD	Standard Deviations
SE	Sleep Efficiency
SREB-1c	Sterol Regulatory Element-Binding Protein 1c
SRI	Sleep Regularity Index
TST	Total Sleep Time
WDR	Weighted Dietary Record
γGT	Gamma-Glutamyl Transferase
